# Factors affecting genetic counseling experiences of foreign residents in Japan: implications for healthcare inclusivity

**DOI:** 10.1007/s12687-025-00833-z

**Published:** 2025-09-30

**Authors:** Kate Nakasato, Moeko Isono, Kazuto Kato

**Affiliations:** Department of Biomedical Ethics and Public Policy, Graduate School of Medicine, The University of Osaka, Suita, Japan

**Keywords:** Genetic counseling, Genomic medicine, Foreign residents, Japan, Diversity, Inclusivity

## Abstract

The rapid development of genomic medicine and simultaneous global diversification of societies present new and complex challenges for healthcare systems worldwide. Medical professionals are now expected to communicate highly complex and evolving genetic information while simultaneously addressing the diverse linguistic, cultural, and social needs of their patient populations. At the center of this effort is the genetic counselor, who must navigate cultural perceptions of genetics, varying levels of health literacy, language barriers, and socioeconomic disparities to deliver equitable and effective care. Research in this area is expanding. However, its global distribution remains uneven and disproportionately concentrated in certain regions. In Japan, where many sectors of society are not yet fully equipped to meet the needs of its increasing migrant population, i.e., foreign residents, research describing the factors that impact their genetic counseling experiences is scarce. To fill this gap, we conducted semi-structured qualitative interviews with ten individuals who have had genetic counseling in Japan for prenatal diagnosis/screening, hereditary cancer, or hereditary monogenic disease. Thematic analysis revealed five factors that impacted their experience with genetic counseling: (1) Japanese language proficiency, (2) genetic literacy, (3) digital health literacy, (4) global family connections, and (5) interactions with medical professionals. These findings not only provide nuance to existing literature but also suggest areas of improvement for the cultural competence training of genetic counselors in Japan and point towards the need for guiding resources at the international level.

**Supplementary Information:**

The online version contains supplementary material available at 10.1007/s12687-025-00833-z.

## Introduction

The rapid development of genomic medicine and the simultaneous global diversification of societies present new and complex challenges for healthcare systems worldwide. Genomic medicine, which tailors disease prevention, diagnosis, and treatment to a person’s individual genetic information, is now being applied in a wide range of fields, including oncology, rare diseases, infectious diseases, pharmacogenomics, and reproductive health (Roth 2019). At the same time, disparities remain regarding how different people and populations engage with and benefit from genomic medicine (National Academies of Sciences et al. 2018). Those who are at particular risk of facing these disparities are migrants and ethnic minorities who experience challenges in accessing healthcare due to (1) linguistic, sociocultural, and socioeconomic conditions that differ from the majority population and (2) the lack of preparedness of the society in which they reside to meet their needs (Wagner 2019). Medical professionals and systems are not only challenged to communicate constantly changing and increasingly complex genetic information to patients, but also to do so while meeting the increasingly diverse needs of their patient populations through cultural competence and policy adaptation, as noted by Joseph et al. (2017) in the context of the United States:

*“The high rates of limited health literacy in the US*,* increasing access of diverse populations to genetic services*,* and growing complexity of genetic information have created a perfect storm. If not directly addressed*,* this convergence is likely to exacerbate health disparities in the genomic age”* (Joseph et al. 2017).

One of the core mediators of this challenge is the genetic counselor. To provide quality genetic counseling that empowers patients and clients to make informed healthcare decisions, genetic counselors must navigate complicated intersections of linguistic and literacy barriers, cultural notions of genetics and health, and socioeconomic disparities in healthcare access (Redlinger-Grosse et al. 2017; Shete et al. 2024). Research in this area is expanding. However, its global distribution remains uneven and disproportionately concentrated in certain regions.

From the United States, there is a wide breadth of research that reflects the country’s long and dynamic history of global immigration and active discourse on diversity, equity, and inclusion (DEI) in healthcare (National Academies of Sciences et al. 2024). Findings from the United States describe a wide range of factors that impact the genetic counseling experiences of racially and ethnically diverse patients such as the genetic literacy of the patient (Scherr et al. 2014), perceptions of illness (Greeson et al. 2001), mistrust towards medical professionals (Cheung et al. 2019), cost of and access to genomic medicine and genetic counseling (Lumpkins et al. 2020), and limited cultural competence of medical professionals (Browner et al. 2003).

Relevant literature also exists from countries outside the United States. Barlow-Stewart et al. (2006) describe the importance of incorporating traditional notions of family, inheritance, and kinship into genetic counseling for Chinese-Australian communities regarding hereditary breast, ovarian, and colorectal cancer. Literature on the barriers preventing indigenous Australians from accessing cancer genetic counseling also describes cultural understandings of illness, cultural inclusivity and accessibility of genetic counseling services, and a lack of awareness of genetic counseling among indigenous communities (Gonzalez et al. 2020). In South Africa, where the cultural and linguistic diversity is reflected by 11 official languages, Zingela et al. (2023) describe how genetic counseling research in the country has focused on communication strategies and capacity building among communities whose languages do not have a well-developed scientific vocabulary to describe genetics concepts. Zayts-Spence (2021) presents evidence from Hong Kong that socioeconomic background and genetic literacy play an underlying role in effective communication among language differences, and that differences in language and culture do not necessarily limit effective genetic counseling because “participants draw on various communication strategies to resolve possible differences and misunderstandings.”

Literature from the aforementioned countries reflects societal contexts in which racial and ethnic diversity is widely recognized and acknowledged. In contrast, notions of national identity and race/ethnicity tend to be more rigid in Japan, and research on the genetic counseling experiences of diverse patient populations is scarce and often lacks the same depth of analysis despite the rapidly increasing number of foreign residents in the country^1^ (Ministry of Justice 2024). A slowly growing amount of literature exists on the barriers to healthcare experienced by foreign residents in Japan (Higuchi et al. 2021; Matsuoka et al. 2022). However, literature on the genetic counseling experiences of foreign residents remains scarce. At present, only three sources exist: two abstracts of poster presentations from academic conferences (Kobori et al. 2019; Mizukami 2023) and one research article written in Japanese, which discusses two genetic counseling cases for foreign residents and reports the experiences of the genetic counselors involved rather than empirical data from the perspective of the patients themselves (Murakami et al. 2009). All three sources identify language and sociocultural differences as the primary challenges in providing genetic counseling. The underlying causes or potential solutions to these challenges, and additional contributing factors, are not explored.

Our study aims to fill this knowledge gap by identifying key factors beyond language and cultural differences that impact the genetic counseling experiences of foreign residents in Japan. In doing so, we aim to inform future efforts toward more inclusive practices in genetic counseling, genomic medicine, and healthcare in Japan. Additionally, this research contributes knowledge from the Japanese context to the global discourse on genetic counseling.

## Methods

This study employs a descriptive qualitative design to identify and describe the factors that impact the genetic counseling experiences of foreign residents in Japan. A descriptive design was chosen for its ability to clearly and concisely summarize experiences as reported by the participants themselves without over interference from the researcher (Hall and Liebenberg 2024). Informational representation was ensured by employing purposive maximum variation sampling based on three key characteristics that were identified as most relevant to the study’s objective: clinical goal of the genetic counseling received, nationality of the participant, and years lived in Japan. Maximum variation sampling was chosen for its ability to cultivate a holistic understanding among participants with significant differences (Bobbitt 2020).

### Data sampling and collection

Participant sampling was carried out through cooperating medical facilities in the Kanto and Kinki regions of Japan, which encompass major prefectures like Tokyo and Osaka. The medical facilities from these regions included university hospitals, outpatient clinics, general hospitals, and advanced treatment hospitals. The research team recruited participants through genetic counselors with experience providing services to foreign residents. These counselors were contacted either directly or via clinical geneticists who had overseen relevant cases. For each potential participant, the genetic counselor who conducted the counseling session facilitated the introduction to the research team. The research team then provided detailed study information and informed consent materials. Participation was confirmed when potential participants contacted the research team after reviewing these materials, indicating their voluntary consent.

Inclusion criteria for the sampling process included the following:

- Over 20 years of age^2^
- Currently living in Japan with foreign resident status^3^
- Received genetic counseling in Japan related to prenatal diagnosis/screening, hereditary cancer, or an inherited genetic condition affecting themselves or their child^4^
- Possess the linguistic competency to be interviewed in English or Japanese.

Potential participants were introduced to the research team and provided information and materials after pre-test or post-test counseling. However, formal recruitment—i.e., when voluntary consent was obtained—and data collection was not carried out until post-test counseling had been completed.

Data was collected through semi-structured qualitative interviews from June to December 2024. Interviews were conducted in English or Japanese based on the preference of the participant. The interview questions were designed to be participant-centered, i.e., to allow open-ended responses and give participants the space to self-identify the experiences that were of greatest importance to them. This was also done to allow a wide range of answers to reflect the diversity among foreign residents in Japan. The interview questions were generated by referencing literature describing similar studies (Joseph and Guerra 2015; Cheung et al. 2019). Interview questions were divided into four parts: (1) pre-counseling experiences and motivations, (2) experiences during counseling, (3) post-counseling experiences and decisions, and (4) overall thoughts regarding their experience with genomic medicine in Japan.

### Data analysis

This study employed thematic analysis (Naeem et al. 2023). Interviews were transcribed in the language in which they were conducted. Phrases that reflected a factor influencing the participant’s experience of genetic counseling were identified as meaning units and manually coded in English. Coding was conducted by the lead author using a combination of deductive methods, based on a pre-established set of codes derived from existing literature, and inductive methods, drawing on meaning units that represented factors not captured previously. A matrix was used to visualize the codes, and similar codes were grouped into sub-themes and themes. Sub-themes were organized into themes that embodied meanings related to the research question of this study, i.e., the factors that impacted the genetic counseling experiences of foreign residents.

Trustworthiness of the analysis was ensured through review, discussion, and refinement of codes, sub-themes, and themes among the lead researcher and co-authors. This collaborative validation process was conducted at the initial, mid-point, and final stages of analysis to enhance consistency and interpretive credibility. Third-party perspectives were also obtained from members of the authors’ research laboratory in Japan.

### Author reflexivity

The lead author of this study is a foreign resident in Japan herself. As a result, the lead researcher recognized the potential for personal biases to influence the study. She explicitly engaged with these biases through critical discussions with the co-authors to ensure that her own experiences did not overshadow the experiences of the participants themselves. Furthermore, the lead researcher’s own experience as a foreign resident provides a unique perspective on the challenges and cultural nuances that participants in the study were likely to encounter. This perspective allowed her to approach the research with empathy and a deeper understanding of the participants’ concerns. The researcher’s identity as a foreign resident herself may also have helped establish trust more easily with the participants, who may have been more willing to share their experiences with someone in a similar position.

The co-authors are Japanese citizens born and raised in Japan. They are also experts in the field of biomedical ethics and have extensive working experience with Japanese genetic counselors. The position of the co-authors contextualized the findings and ensured balance of perspectives with the lead author. Like the lead author, the co-authors recognized the potential for their own biases to influence their impression of the experiences of foreign residents and thus impact the study. They engaged with these biases through critical discussions with the lead author to ensure that their perceptions of the experiences of foreign residents did not overshadow the actual voices and experiences of the participants themselves.

## Results

### Study participants and cases

We received consent to participate from 10 participants, which made up eight cases of genetic counseling.^5^ Five of these cases were related to prenatal screening, while two cases were related to hereditary cancer, and one case was related to an inherited genetic condition.^6^ All participants received genetic counseling in 2024 from a certified genetic counselor and had no previous experience with genetic counseling or genomic medicine in their home countries (Table 1).

**Table 1:** Participant characteristics

Case	Age	Gender	Nationality	Years Lived in Japan	Genetic Test	Test Result	Japanese Level	Counseling Language	Interview Language	Prior Knowledge of Genetics
1	30s	F	France	3	NIPT	-	N4	English	English	N
	30s	M	France	3			N4			N
2	30s	F	Iran	4	NIPT	-	N4	English	English	N
3	30s	F	China	12	NIPT	-	N2	Japanese	Japanese	Y
4	30s	F	China	6	NIPT	-	N3	Japanese	Japanese	N
5	30s	F	Poland	1	NIPT	-	N4	Japanese with Translator	English	Y
6	30s	F	Brazil	7	PALB2	+	N3	Japanese	English	Y
7	60s	M	USA	20+	BRCA1/2	-	N4	English	English	F
8	30s	F	China	16	SBDS	+	N2	Japanese	Japanese	F
	30s	M	China	11	SBDS	+	N2			N

*M* male, *F* female, *NIPT* non-invasive prenatal testing, *PALB2* PALB2 gene sequencing, *BRCA1/2* BRCA1/2 gene sequencing, *SBDS* shwachman-bodian-diamond syndrome gene sequencing, *‘+’* positive result, *‘-’* negative result, *Y* yes, prior knowledge of genetics, *N* no, no prior knowledge of genetics, *F* no prior knowledge of genetics but received knowledge from a family member

The linguistic competence (Japanese ability) of each participant was determined using the five levels of the Japanese Language Proficiency Test (JLPT) as a reference, where N5 represents the lowest linguistic competence and N1 represents the highest linguistic competence (Japan Foundation and Japan Educational Exchanges and Services 2012). The linguistic competence levels of the participants were either directly self-reported by the participants based on JLPT levels or, in cases where participants had not taken the JLPT, determined by comparing the participants’ descriptions of their Japanese level with the JLPT’s descriptions of each level.^7^

#### Case 1

Case 1 included a husband and wife in their 30 s from France who received genetic counseling regarding prenatal screening. At the time of the interview for this study, they had lived in Japan for three years and had a beginner (N4) level of Japanese language proficiency. They chose to be interviewed for this study in English. While English was not their first language, they were able to participate in the interview without significant communication barriers. They did not report any prior knowledge of genetics. Both husband and wife attended genetic counseling and the interview for this study together. They received genetic counseling as a requirement for non-invasive prenatal testing (NIPT), which they sought after receiving abnormal results from a quad screen. The medical institutions they attended provided genetic counseling in English, accompanied by visual materials. The test result was negative.

#### Case 2

Case 2 included a woman in her 30 s from Iran who received genetic counseling regarding prenatal screening. At the time of the interview for this study, she had lived in Japan for four years and had intermediate (N3) Japanese language proficiency. She chose to be interviewed for this study in English. While English was not her first language, she was able to participate in the interview without significant communication barriers. She did not report any prior knowledge of genetics. Her husband, also from the Iran, attended genetic counseling with her but was unavailable for an interview for this study. They received genetic counseling as a requirement for NIPT, which they sought due to the woman’s condition of thalassemia beta. While the medical institution she attended had genetic counselors who could offer genetic counseling in English, they received genetic counseling in Japanese with the aid of visual materials in English due to the lack of availability of English-speaking genetic counselors at the time. The test result was negative.

#### Case 3

Case 3 included a woman in her 30 s from China who received genetic counseling regarding prenatal screening. At the time of the interview for this study, she had lived in Japan for over ten years and had advanced (N2) proficiency in the Japanese language. She chose to be interviewed for this study in Japanese. She reported prior knowledge of genetics due to her occupation in the medical field. Her husband, also from China, attended genetic counseling with her but was unavailable for an interview for this study. They received genetic counseling as a requirement for NIPT, which they sought due to the age of the woman. While the medical institution they attended did not have clinical geneticists or genetic counselors who could offer genetic counseling in English, they received genetic counseling in Japanese with no significant language barrier. The test result was negative.

#### Case 4

Case 4 included a woman in her 30 s from China who received genetic counseling regarding prenatal screening. At the time of the interview for this study, she had lived in Japan for six years and had intermediate (N3) Japanese language proficiency. She chose to be interviewed for this study in Japanese. She did not report any prior knowledge of genetics. Her husband, who was also from China but had a higher Japanese language proficiency than his wife, accompanied her during genetic counseling, serving as both a client and a translator; however, he was not available for an interview for this study. They received genetic counseling as a requirement for NIPT, which they sought after receiving abnormal nuchal translucency (NT) scan results. While the medical institution they attended had genetic counselors who could offer genetic counseling in English, the couple’s Japanese language ability was sufficient and stronger than their English language ability to receive genetic counseling in Japanese. The test result was negative.

#### Case 5

Case 5 included a woman in her 30 s from Poland who received genetic counseling regarding prenatal screening. At the time of the interview for this study, she had lived in Japan for one year and had a beginner (N4) level of Japanese language proficiency. She chose to be interviewed for this study in English. While English was not her first language, she was able to participate in the interview without significant communication barriers. She reported prior knowledge of genetics due to majoring in biology during university. Her husband, who was Japanese, accompanied her during genetic counseling as both a client and a translator for his wife. They received genetic counseling as a requirement for NIPT, which they sought due to the presence of a family member with Down syndrome. While the medical institution they attended had genetic counselors who could offer genetic counseling in English, they received genetic counseling in Japanese because the husband was present as a translator. The test result was negative.

#### Case 6

Case 6 included a woman in her 30 s from Brazil who received genetic counseling regarding hereditary cancer. At the time of the interview for this study, she had lived in Japan for seven years and had intermediate (N3) Japanese language proficiency. She chose to be interviewed for this study in English. While English was not her first language, she was able to participate in the interview without significant communication barriers. She reported prior knowledge of genetics due to her occupation in the medical field. Her husband, who was from the UK, did not attend genetic counseling with her. She sought genetic testing and genetic counseling after a pathogenic variant linked to breast cancer was identified within her family, which was due to a family member’s participation in a breast cancer study in her home country. Unlike her family members, she did not yet have symptoms of breast cancer. However, genetic testing was recommended by her family in her home country. While the medical institution she attended offered Japanese-English translation services, she received genetic counseling in Japanese due to convenience and confidence in her ability to communicate in Japanese. The test result was positive.

#### Case 7

Case 7 included a man in his 60 s from the United States of America who received genetic counseling regarding hereditary cancer. At the time of the interview for this study, he had lived in Japan for over 10 years and had a beginner (N4) level of Japanese language proficiency. He chose to be interviewed for this study in English, which was his first language. He did not report any prior knowledge of genetics but indicated that he had connections with an overseas family member who had prior knowledge of genetics due to their occupation in the medical field. His wife, who was Japanese, did not attend genetic counseling with him. His physician in Japan recommended him genetic counseling due to a family history of prostate cancer. At the time of the interview for this study, he had been diagnosed with prostate cancer roughly 10 years prior and was in remission. The medical institution he attended had clinical geneticists who provided genetic counseling in English. The test result was negative.

#### Case 8

Case 8 included a husband and wife in their 30 s from China who received genetic counseling regarding the possibility of a genetic condition. At the time of the interview for this study, both the husband and wife had lived in Japan for over 10 years and had advanced (N2) proficiency in the Japanese language. They chose to be interviewed for this study in Japanese. They did not report any prior knowledge of genetics, but indicated that they had connections with an overseas family member who had prior knowledge of genetics due to their occupation in the medical field. Both husband and wife received the same genetic test and attended genetic counseling and the interview for this study together. They received genetic counseling as a requirement for genetic testing, which they sought after the detection of severe fetal abnormalities led them to terminate a previous pregnancy. While the medical institution they attended had clinical geneticists who could offer genetic counseling in English, the couple’s English language abilities were weaker than their Japanese language abilities and their Japanese language ability was sufficient to receive genetic counseling in Japanese. The test results were positive.

### Key themes

Thematic analysis yielded 5 themes and 11 sub-themes. The 5 themes indicate what factors impacted the genetic counseling experiences of the participants. The 11 sub-themes indicate how these factors limited or enabled successful genetic counseling. In this study, successful genetic counseling is defined as access to counseling that effectively communicates the necessary information for participants to make informed decisions afterward (Table 2).

**Table 2:** Themes and Sub-themes

Themes	Sub-themes
Japanese Language Proficiency	Limited Comprehension
	Limited Choice of Medical Institutions
Genetic Literacy	Compounded Language and Genetic Literacy Limitations
	Overcoming Language Limitations with Genetic Literacy
Digital Health Literacy	Overcoming Language Limitations with Online Resources
	Overcoming Genetic Literacy Limitations with Online Resources
Global Family Connections	Motivation to Seek Genetic Testing/Counseling
	Source of Genetic Literacy
Interactions with Medical Professionals	Visual Aids to Promote Communication
	Relief from Positive Experiences
	Anxiety from Negative Experiences

### Japanese language proficiency

Low Japanese language proficiency 1) limited participants’ comprehension during genetic counseling, and 2) limited participants’ choice of medical institutions. Case 5 described how the significance of genetic counseling was lessened for her because she could not understand what was being said in Japanese. She also noted that a hospital’s acceptance of participants without Japanese language proficiency was the deciding factor to choosing a medical institution.

“I didn’t feel like [genetic counseling] was very needed… since it was in Japanese, it was difficult for me to understand anyway.” (Case 5, N4).

“I have read online, sometimes, in terms of reviews, for example on Google Maps, of different clinics or hospitals where foreigners would say that they would not be attended because they cannot communicate very well in Japanese. As a foreigner, it is quite a challenge to have to face that. The deciding factor (to choosing a hospital) was that they welcome foreigners or English speakers and that I could be accompanied by my husband to facilitate the process.” (Case 5, N4).

### Genetic literacy

Low genetic literacy worsened participants’ comprehension limitations already caused by low Japanese language proficiency. Case 5 stated that even though her Japanese husband accompanied her as a translator, she did not feel confident that he would be able to translate the information correctly to her due to his lack of knowledge of genetics.

“I was so relieved when [the NIPT test result] was negative. But all [of my concerns did] not just fade away by just [the negative test result], because genetics is so complicated, and I don’t know anything about it…” (Case 2, N4).

“My husband accompanied me, but he doesn’t really have… he’s not knowledgeable about [genetics] at all in general, and even less so in English. I think that could be a barrier, as he could not convey to me very well what was being said.” (Case 5, N4).

On the other hand, high genetic literacy was used to improve comprehension despite limited Japanese language proficiency. Participants without an advanced command of Japanese (N4 to N3) reported being able to comprehend the content of genetic counseling in Japanese due to their familiarity with genetics.

“I understand some Japanese, and definitely knowing the topic [of genetics] also made it easier to understand what was being said. So in many situations, I didn’t feel like I needed the translation.” (Case 5, N4).

“I already knew the concept of genetic counseling, because I’m a researcher, so I also studied genetics at some level. And so it was relatively easy for me to find online… [The genetic counselor] had graphs and statistics and a computer presentation of what happens… It was very detailed… I think [the information presented during genetic counseling] was easy to understand, but I’m used to looking at graphs. It’s very useful to have those graphs, but for me it’s easy because I’m used to looking at this type of information.” (Case 6, N3).

### Digital health literacy

Digital health literacy was used by participants to navigate online resources to overcome 1) low Japanese language proficiency and 2) low genetic literacy. Case 1, Case 2, and Case 5 accessed online resources written in English or their native languages. Case 3, who had both high Japanese language proficiency and high genetic literacy, sought more detailed information about the lived experiences of people born with genetic conditions through online videos.

“Of course, we also looked at the information on the internet [before genetic counseling]. We found French websites, and then, plus the video [during genetic counseling], it was very clear.” (Case 1, N4).

“[The genetic counseling session was] a little scientific to me, but I tried myself to understand it in my own way, because after that I just research it myself.” (Case 2, N4).

“[I was] kind of me expecting that the [genetic counseling] would be in Japanese, so I just prepared myself beforehand and checked the information in English or in Polish from reliable sources, which is why the [genetic counseling] was rather short… I just searched it online. I wanted to educate myself, and I was relying on the Polish or American, or UK sites.” (Case 5, N4).

“I could understand [the genetic counseling] in Japanese. Chromosome 21 is the easiest to understand, but maybe 13 or… there are many, aren’t there? When I heard about those, I thought ‘Oh, yes, there are such things,’ because I’ve studied about them in school… I did a lot of research [at home] on the internet, and what I was looking for was what would happen to the children if they had a chromosomal abnormality. I was looking at videos and other things… There are a lot of [people with genetic conditions or chromosomal abnormalities] who are working, just like normal working people. I watched these kinds of videos. I didn’t watch scientific videos or anything like that. I watched the videos [about people’s lived experiences], and then I looked at the comments below and decided whether or not I needed to undergo the test. (Case 3, N2, quote translated from Japanese)

### Global family connections

Knowledge from overseas family members of participants 1) acted as a motivator to seek genetic counseling/testing, and 2) promoted patient comprehension despite low genetic literacy. In Case 6, the development of genomic medicine in the participant’s home country led her to get genetic testing and counseling herself and ultimately receive a diagnosis. In Case 8, where the participant terminated a previous pregnancy due to severe fetal abnormalities, a family member who was a medical professional advised her to get genetic testing and ultimately receive a diagnosis.

“My [family member] joined research in Brazil about breast cancer at a Brazilian university, and at the university, that research, part of that research involved genetic testing, and they found a mutation. My [family member] carries a mutation that is highly associated with breast cancer. And then my [other family member] was tested as well. She also has the mutation. My [family member’s] siblings also got tested. Two of them also had the mutation. And then I was highly advised to get tested as well by my family and their doctors back in Brazil.” (Case 6, N3).

“My [family member] is an OBGYN (Obstetrician and Gynecologist), so she gave me all kinds of advice. She said, ‘If the fetus had such a serious condition, shouldn’t you get a genetic testing?’” (Case 8, N2, quote translated from Japanese).

“Actually my [family member] is… she’s kind of the mover in the family, the one who is up on the most recent research… [My family member] sent me a few research articles [about BRCA 1/2].” (Case 7, N4).

### Interactions with medical professionals

Visual aids were used by genetic counselors to promote comprehension despite the patient’s low Japanese language proficiency. Case 1 described how the use of a video to explain NIPT during genetic counseling made it easier to understand. Case 4 described how written handouts were helpful because of the use of Chinese *kanji* characters in written Japanese. Case 6 explained how the doctor’s spoken explanations were vague and unclear; she did not understand the results of the case until she was provided a written handout of the results.

“We had a video of 19 minutes. It was very clear. I think I understood better with the video. The video was very clear because there were images, and it’s easier than speaking, because we will miss many information [when speaking].” (Case 1, N4).

It was okay because there were written materials. Just listening [to the explanation] is… During spoken explanations, my husband is there. My husband’s Japanese is good.

Researcher: “the written materials were easy to understand because of the Kanji (Chinese characters)?”

“Yes, that’s right.” (Case 4, N3, quote translated from Japanese).

“It felt unclear to me what the result was until I actually looked at the paper myself. He put the paper down and it was like obviously all in Japanese, and I’m listening to him and it’s… the way he said it. I don’t remember the exact words now because it was in Japanese… but he did say *arimashita* (the mutation is there) I think at some point. But it wasn’t clear.” (Case 6, N3).

Participants also described how they felt 1) relief after positive communication experiences with medical professionals and 2) anxiety after negative communication experiences with medical professionals. Participants tended to emphasize how information was conveyed to them, particularly the patience of the medical professional and how much time was taken to explain the information, rather than what information was conveyed or in what language the information was conveyed. Here, we define positive communication experiences as interactions in which medical professionals took measures to address the participants’ communication needs, e.g., taking extra time, demonstrating patience, and adjusting explanations to match the participant’s level of Japanese proficiency. In contrast, we define negative communication experiences as interactions where such measures were lacking. For example, Case 6 described a positive experience, while Case 4 described both types of experiences:

“The moment [the genetic counselor] realized that I was not Japanese, she used very simple Japanese words, and I felt confident. No one seemed troubled to take a few extra minutes to explain to me, and nobody seemed troubled with the fact that I couldn’t fluently speak Japanese. It was fine.” (Case 6, N3).

“I first went to a clinic near my house. At that time, there was a problem with my NT test results, but the doctor there did not give me a detailed explanation. He just told me the results were not good and said, ‘I want you to go to [Hospital A] and get NIPT.’ That was it. What is NIPT? I don’t know. I wished the doctors at the clinic and at the hospital would’ve explained it to me. [The doctor at the clinic near my house] took only 10 minutes… and I didn’t understand. I didn’t understand it at all. So that day, when I got home, I cried… I was scared. [But at Hospital A], the doctors were all very kind. I am calmer in an environment like that. Their voices are kind. Their smiles, too. I’m a foreigner and don’t really understand Japanese, which makes me nervous. But [at Hospital A], everyone explained things to me kindly and I felt relieved.” (Case 4, N3, quote translated from Japanese).

Case 1 also pointed how awareness of the stresses foreign residents in Japan can face would facilitate positive communication experiences.

“[I want doctors to be] more sensitive to the fact that foreigners in Japan can undergo a lot of stress [from] discrimination. Maybe if they know that we are under this kind of stress, they can better understand and communicate [with us].” (Case 1, N4).

## Discussion

Findings from our study revealed five factors that may impact the genetic counseling experience of foreign residents in Japan: (1) Japanese language proficiency, (2) genetic literacy, (3) digital health literacy, (4) global family connections, and (5) interactions with medical professionals. These factors limited or enabled successful genetic counseling, depending on the patient’s individual characteristics and circumstances.

Japanese language proficiency was identified by participants as a limiting factor to successful genetic counseling. Our findings align with existing literature from Japan (Murakami et al. 2009; Kobori et al. 2019; Mizukami 2023) and add further insight by specifying limited access to and comprehension during genetic counseling as potential outcomes.

Genetic literacy was identified by participants as a limiting or enabling factor, depending on the patient’s proximity to genetics (e.g., due to occupation, knowledge from a family member, personal experience, etc.) and Japanese language proficiency. Some participants described how their familiarity with genetics and related terminology facilitated their understanding during genetic counseling despite language barriers. This suggests that in the case of genetic counseling for foreign residents in Japan, patient comprehension during genetic counseling lies at the intersection of language proficiency and genetic literacy. Our findings add nuance to the aforementioned literature from Japan, which often treats language proficiency as a standalone factor. They also contribute to broader discussions on the intersection of language and literacy. For example, studies from South Africa (Zingela et al. 2023) and Australia (Vass et al. 2011) emphasize that genetic literacy must be addressed alongside the absence of equivalent vocabulary in patients’ native languages or beliefs of health and illness that do not follow a biomedical worldview. While this issue pertains more to linguistic vocabulary than to language proficiency, it underscores the critical role that the interplay between language and literacy plays in shaping patient understanding during genetic counseling. Furthermore, the experiences of some participants from this study suggest that low genetic literacy combined with low Japanese language proficiency can create a compounded limitation to comprehension. These findings align with existing literature from other healthcare fields that highlight how these compounded limitations can contribute to health inequalities (Poureslami et al. 2011; Hughson et al. 2018; Ugas et al. 2023). The confusion and anxiety caused by this limitation impact the patient’s well-being and could be exacerbated further if the patient experiences negative interactions with medical professionals, such as with Case 4 in our study. This underscores the importance of cultural competence training for medical professionals to meet patients’ linguistic and literacy needs.

Digital health literacy was utilized by participants to facilitate comprehension in the case of low language proficiency and/or genetic literacy. Participants described how they navigated online resources written in English or their native languages after genetic counseling sessions to clarify information they did not understand. The importance of digital health literacy in general is recognized in existing literature in terms of navigating online health services (e.g., patient portals) (Bywall et al. 2025). The importance of online platforms and digital tools have also been recognized, particularly in the field of rare disease (Chang et al. 2025). Our findings align with existing knowledge on the importance of digital health literacy and digital resources and add to existing knowledge by pointing out the importance of these resources to compensate for communication and literacy barriers, specifically in the field of genetic counseling. Our findings also underscore the need for reliable online resources available in a diverse range of languages. In Japan, the Committee for the Accreditation System of Prenatal Testing (*shussei zen shindan ninshou seido-tou unei iinkai*) currently provides information about NIPT in English and Chinese (Committee for the Accreditation System of Prenatal Testing, Japan Society of Obstetrics and Gynecology 2023). However, as immigration continues to rise in Japan, there is a need to expand these translations to include other genetic tests and other languages spoken in countries with growing immigrant populations, such as Vietnam, Indonesia, Myanmar, and Thailand. Furthermore, our findings point to the need for internationally coordinated guidelines to help mitigate confusion stemming from cross-border differences in medical systems and policies surrounding genomic services. Such guidelines would assist patients who access online health information in their native language from their country of origin, which may not reflect the healthcare context of the country in which they currently reside.

A family member in the participants’ home country was sometimes the catalyst for participants to seek genetic counseling. A family member with high genetic literacy was also sometimes utilized by participants to facilitate comprehension if the participant themselves had low genetic literacy. Our findings highlight the importance of social connections as an information source and align with existing literature that recognizes social support as especially important for migrant communities that face language barriers and discrimination (Kim et al. 2015; Matsuoka et al. 2022). Our findings also point to the global nature of genomics, especially in the modern era where globalization moves people across borders and communication can be maintained regardless of distance. Looking ahead, cases like Case 6 in our study may become increasingly common. In this case, diagnosis was only made possible through communication with family members who underwent genetic testing in the participant’s country of origin. As transnational family networks and global access to genetic testing expand, such cross-border exchanges of genetic information may play a more prominent role in clinical outcomes.

Finally, participants identified certain interactions with medical professionals as either limiting or enabling factors to successful genetic counseling. Use of visual aids such as written materials, videos, and diagrams were described as helpful to understand the information during genetic counseling despite low Japanese language proficiency. This aligns with existing literature, which shows that visual aids can increase understanding in patients with language barriers (Hazimeh et al. 2023). The emotional state of participants was also impacted by interactions with medical professionals, causing either relief or anxiety and contributing to the participants’ overall genetic counseling experience. These interactions were associated with how information was communicated to participants, i.e., speaking slowly to meet the participant’s level of Japanese language proficiency, treating the participant kindly to ease worries that the medical professional will be impatient or discriminatory because of the participant’s identity as a foreign resident, taking sufficient time to clearly explain test results, etc. These findings emphasize the importance of cultural competence training, which is defined in the context of healthcare as “the ability of systems to provide care to patients with diverse values, beliefs, and behaviors, including tailoring delivery to meet patients’ social, cultural, and linguistic needs” (Truong et al. 2014). According to Okamoto et al. (2019), competence in culturally sensitive care in healthcare has been included in the Japanese medical school curriculum since 2001 and in the Japanese nursing school curriculum since 2018 (Okamoto et al. 2019). In various Master’s programs for genetic counseling in Japan integrate roleplay modules on patients from “diverse values and social backgrounds” (Jinkei University 2024). However, the specific content of this training is unclear.

## Conclusion

Our study identified five factors that may impact the genetic counseling experiences of foreign residents in Japan: Japanese language proficiency, genetic literacy, digital health literacy, global family connections, and interactions with medical professionals. The main limitations of our study include sample size and sampling inclusion criteria. The relatively small sample size limits our ability to generalize findings to all foreign residents in Japan. Furthermore, the sampling method also limits our ability to capture the experiences of those who were unable to access genetic counseling or those who received genetic counseling but did not possess the English or Japanese language proficiency to participate in this study. At the same time, our findings add nuance to existing literature from Japan by revealing factors beyond already recognized language and cultural differences. It also points to ways in which patients use various resources to navigate language and genetic literacy challenges, which have implications for not only future cases of genetic counseling for foreign residents, but also cases of genetic counseling for Japanese patients and patients of minority populations in other countries. Future studies that include larger sample sizes and participants with various language proficiencies may further validate our findings. Future studies that do not generalize foreign residents in Japan as a single category but instead describes the experiences of specific groups based on factors such as ethnicity, socioeconomic status, age, gender, or religion would further deepen understanding in this area of research.

Ultimately, strengthening accessibility and inclusivity requires a shift toward recognizing and addressing the diverse and intersecting factors that shape each patient’s experience. As genomic technologies become increasingly embedded in global healthcare systems, tackling issues of diversity, inclusivity, and equity in genetic counseling must be a priority to ensure that the benefits of genomics are distributed fairly and responsibly. For Japan, this may involve expanding cultural competence training for genetic counselors and other healthcare professionals. Existing training for genetic counselors in Japan would benefit from more advanced and nuanced cultural competence education. It should go beyond basic *awareness* of linguistic and cultural differences and include structured opportunities to develop skills in cross-cultural communication, analyzing cases from an intersectionality lens, and case-based learning that reflects the growing diversity of patient populations. Diversification of Japan’s medical and healthcare workforce to reflect the cultural and linguistic diversity of its patient population may also strengthen the quality of healthcare provision, including but not limited to genomic medicine and genetic counseling. At the global level, this may include the development of comprehensive guidelines for genetic counselors at the international level, which would function similarly to existing international guidelines in genomics that serve as a high-level, evidence-based framework that countries can adapt to their own policies and sociocultural contexts. Regardless of context, a commitment to inquiring the causes of and solutions to the barriers to inclusive genetic counseling and genomic medicine is essential for a future in which all individuals, regardless of language, literacy, nationality, or culture, can benefit from advances in the field of genomics.

## Supplementary Information

Below is the link to the electronic supplementary material.

Supplementary Material 1 (PDF 557 KB)

## Data Availability

The author wishes to maintain the confidentiality of the research data in order to safeguard the individual privacy of the respondents. All pertinent information and resources are stored in compliance with the ethical protocols of the Ethical Review Board at The University of Osaka Hospital (Approval Number 23490).

## Footnotes

1 As of June 2024, 3,588,956 foreign residents were registered in Japan. This number represents an increase of approximately half a million in just the previous two years.

2 While the age of adulthood is 18 in many countries, Japan legally recognized adulthood as 20 years of age until 2022—just two years prior to the implementation of this study. Given the sensitive nature of the research, which involves personal and health-related experiences of genetic counseling, we adopted a conservative approach by adhering to the former age threshold. This decision was made to ensure that all participants were unquestionably capable of providing fully informed consent.

3 While nationality was one of the guiding characteristics used in the maximum variation sampling for this study, it was not included as a formal inclusion criterion because nationality alone does not reflect key contextual factors such the clinical goal of the genetic counseling received, language proficiency, or healthcare needs, all of which are more directly relevant to the research objectives.

4 While information about the study was shared with potential participants who had received genetic counseling due to their child’s inherited condition, these individuals did not elect to participate. As a result, all participants in this study were probands themselves.

5 In two cases, husband and wife received genetic counseling together, and both participated in this study.

6 While the distribution amongst the clinical goals of each genetic counseling case is not distributed equally, we believe it roughly reflects the current state of genetic counseling provision in Japan based on relevant data (Japan Agency for Medical Research and Development (AMED) 2016).

7 Descriptions of each JLPT level are as follows. N5 (Beginner): The ability to understand some basic Japanese. N4 (Beginner): The ability to understand basic Japanese. N3 (Intermediate): The ability to understand Japanese used in everyday situations to a certain degree. N2 (Advanced): The ability to understand Japanese used in everyday situations, and in a variety of circumstances to a certain degree. N1 (Advanced): The ability to understand Japanese used in a variety of circumstances.
