# Supplementary material for: Factors affecting genetic counseling experiences of foreign residents in Japan: implications for healthcare inclusivity
Source: J Community Genet. 2025 Sep 30;16(6):839–51. doi: 10.1007/s12687-025-00833-z (PMC12569224; doi:10.1007/s12687-025-00833-z)
Supplement: Supplementary file 1 — Supplementary Material 1 (PDF 557 KB) [file 12687_2025_833_MOESM1_ESM.pdf]

2024 年 02 月 09 日作成　第 1 版

Equity in Genomic Medicine in Japan: An Interview Survey of Foreign Residents' Experiences with Genetic Counseling

（日本における遺伝医療の公平性：遺伝カウンセリングを中心とした在留外国人の経験に関するインタビュー調査）

Interview Questions

（質問票）

Basic Information（基本情報）

1. Age (20-29、30-39、40-49、50-59、60+)
   年齢（20 代、30 代、40 代、50 代、60 代以上）
2. Nationality
   国籍
3. Status of residence in Japan
   在留資格
4. Length of stay in Japan
   在留期間
5. Where you received genetic counseling
   遺伝カウンセリングを受けた都道府県
6. About your family's residence
   ご家族の居住国について（日本、母国など）

Main Questions（主な質問）

1. Please tell us about your experiences leading up to receiving genetic counseling.
   遺伝カウンセリングに至った経験について教えてください。
   a. For what reason did you receive counseling? If possible, we would also like to know about the condition/disease for which you received counseling. Other tests? どんなきっかけで遺伝カウンセリングを受けましたか？ もしよろしければ、どのような疾患について相談したか教えていただけますか？
   b. Were you referred by a physician? Or did you search for counseling by yourself?
      医師から紹介されましたか？自分で受けたいと思ってカウンセラーを探しましたか？
      i. If you were referred by a physician, what was your initial reaction?

医師に紹介された場合、紹介された時にどう思いましたか？

ii. If you searched for counseling by yourself, please tell us about your search process.
カウンセラーを自分で探した場合、そのプロセス（探し方）について教えてください。

c. What expectations or concerns did you have about the experience? Have you received genetic counseling in your home country?
遺伝カウンセリングを受けた前に、どのような期待や心配を感じましたか？母国で遺伝カウンセリングを受けたことがありましたか？

2. Please tell us about your experiences during counseling.
遺伝カウンセリングを受けていた時の経験について教えてください。

   a. How was information explained to you?
   情報はどのふうに説明されましたか？

      i. Was the information easy to understand?
      カウンセリングによる情報はわかりやすかったですか？

      ii. Was the information helpful to make decisions?
      カウンセリングによる情報は役に立ちましたか？

   b. How did you feel about your interactions with the genetic counselor or other medical professionals?
   遺伝カウンセラーと他の医療従事者とのやり取りについてどう感じましたか？

3. Please tell us about your experiences after counseling.
カウンセリングの後の経験について教えてください。

   a. How did counseling affect your decisions afterwards?
   カウンセリングはその後の行動にどのような影響を与えましたか？

   b. How did you share information from counseling with your family or other people around you?
   カウンセリングでもらった情報は家族、親戚、周りの人に伝えましたか？

4. Please tell us your overall thoughts about your experiences receiving genomic services (e.g. genetic counseling, genetic testing, etc.) in Japan.
日本での遺伝医療（遺伝カウンセリング、遺伝子検査など）に対する全体的な意見について教えてください。

   a. Which moments during the process left a particularly strong impression on you?
   全体の経験の中で特に印象に残ったことは何ですか？

   b. What aspects of genetic counseling in Japan were particularly helpful or challenging?

日本での遺伝カウンセリングで、特に役に立ったこと、あるいは特に困難になったことは何でしたか？

c. How should genetic counseling in Japan be improved?
日本における遺伝カウンセリングはどのように改善されるべきだと思いますか？

d. What do you want genetic counselors in Japan to know or understand more?
日本の遺伝カウンセラー、または遺伝医療に関わる医療従事者にもっと知ってほしい、あるいは分かってほしいことは何ですか？
